# Anisotropic lattice response induced by a linearly-polarized femtosecond optical pulse excitation in interfacial phase change memory material

**DOI:** 10.1038/srep19758

**Published:** 2016-01-25

**Authors:** Kotaro Makino, Yuta Saito, Paul Fons, Alexander V. Kolobov, Takashi Nakano, Junji Tominaga, Muneaki Hase

**Affiliations:** 1 Nanoelectronics Research Institute, National Institute of Advanced Industrial Science and Technology (AIST), Tsukuba Central 5, 1-1-1 Higashi, Tsukuba 305-8565, Japan; 2 CREST, JST, 4-1-8 Honcho, Kawaguchi, Saitama, 332-0012, Japan; 3 Division of Applied Physics, Faculty of Pure and Applied Sciences, University of Tsukuba, 1-1-1 Tennodai, Tsukuba 305-8573, Japan

## Abstract

Optical excitation of matter with linearly-polarized femtosecond pulses creates a transient non-equilibrium lattice displacement along a certain direction. Here, the pump and probe pulse polarization dependence of the photo-induced ultrafast lattice dynamics in (GeTe)_2_/(Sb_2_Te_3_)_4_ interfacial phase change memory material is investigated under obliquely incident conditions. Drastic pump polarization dependence of the coherent phonon amplitude is observed when the probe polarization angle is parallel to the *c*–axis of the sample, while the pump polarization dependence is negligible when the probe polarization angle is perpendicular to the *c*–axis. The enhancement of phonon oscillation amplitude due to pump polarization rotation for a specific probe polarization angle is only found in the early time stage (≤2 ps). These results indicate that the origin of the pump and probe polarization dependence is dominantly attributable to the anisotropically-formed photo-excited carriers which cause the directional lattice dynamics.

Ultrafast control of a lattice system realized by direct excitation of infrared-active phonon modes with infrared and THz optical pulses as well as indirect pathways via electron-phonon interactions and ionic Raman scattering^1^ can be key techniques for coherent manipulation of structure in condensed matter. In correlated electron materials, excitation of phonons is one of the most promising pathways for controlling the electronic degrees of freedom via the interplay between phonons and electrons^2^,^3^,^4^. Furthermore, structural change can be achieved by phonon-induced deformation of the lattice^5^,^6^,^7^,^8^. For more sophisticated lattice control, the direction of lattice deformation should be optimized and one prospective approach is the formation of anisotropic carrier distributions which may cause directional phonon oscillation.

Coherent phonon spectroscopy is a time-resolved experimental tool to explore photo-induced optical phonons that oscillate in the THz frequency range. In opaque materials, coherent optical phonons can be driven by three well-known mechanisms, namely, impulsive stimulated Raman scattering (ISRS)^9^,^10^, displacive excitation of coherent phonons (DECP)^11^, and ultrafast screening of the space-charge (SC) field^12^. Recent theoretical studies have succeeded in the computational treatment of not only the isotropic but also the anisotropic contribution to the photo-induced driving force of coherent phonons and have revealed new quantitative insights about the optical response of the lattice^13^. These results suggest that excitation of anisotropic carrier distributions can drive coherent atomic motion beyond the framework of Raman scattering.

Here, we report on anisotropically-driven coherent optical phonons which are excited and detected by linearly-polarized optical pulses in (GeTe)_2_/(Sb_2_Te_3_)_4_ multi-layered film (superlattice), which are used for interfacial phase change memory (iPCM)^14^. Phase change memory has been commercialized in the form of both optical disks and nonvolatile electrical memories^15^. Among the various kinds of phase change memory, iPCM is one of the most promising candidates for the next-generation of non-volatile electrical memory and also has received considerable attention due to its topological insulating nature^16^,^17^, which arises from its multi-layered structure^18^,^19^. Ultrafast manipulation of iPCM structure is relevant to high-speed data storage and highly desirable for the future of large-volume data processing. Previously, it was found that anisotropic phonon response is related to a phase transition in cubic iPCM material, which is different from the currently-used hexagonal iPCM^7^. Recently, the hexagonal iPCM material is mainly investigated for data storage applications because of good repeatability of SET-RESET cycles caused by small volume change upon phase change operation in contrast to the cubic iPCM material. The hexagonal iPCM is expected to present more significant anisotropy in coherent phonon response than the cubic iPCM material because the hexagonal structure is likely to emphasize the contrast between the basal plane and the stacking axis (*c*–axis) directional phonon response. Thereby, we investigated the polarization dependence of coherent phonons in hexagonal iPCM material.

## Results

**Figure 1:**
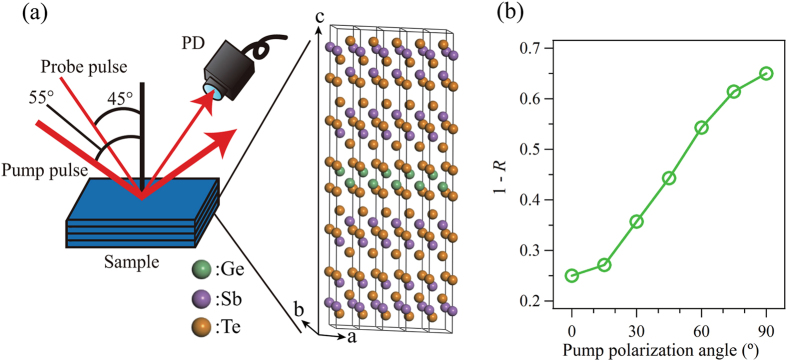
Optical pump-probe setup and polarization dependent 1 − *R* of the pump pulse. (**a**) Illustration of the obliquely incident pump-probe measurement setup. A structural model of the sample is shown in a magnified view. The green, purple and orange balls indicate Ge, Sb and Te atoms, respectively. The intensity of the reflected probe pulse was detected by a photo diode (PD). (**b**) The polarization dependence of the 1 − *R* of the pump pulse.

Coherent phonon spectroscopy measurements were carried out for various polarization angles of pump and probe pulses. Figure 1(a) shows a schematic illustration of the experimental setup and a structural model of the sample. The transient reflectivity change of the probe pulses (Δ*R*/*R*) was recorded as a function of the pump-probe time delay. The polarization angles of pump (*θ*_pump_) and probe (*θ*_probe_) pulses were varied between *s*-polarization (0°) and *p*-polarization (90°). Hereafter, the combination of *θ*_pump_ and *θ*_probe_ is represented as (*θ*_pump_, *θ*_probe_). Due to the oblique incident configuration, the electrical field of *s*-polarized pulse exclusively oscillates parallel to the in-plane (*a* − *b* plane of crystal) direction of the sample while that of *p*-polarized pulse has a component along the out-of-plane (*c*–axis) direction as well. Figure 1(b) shows the polarization dependence of the optical absorption of the pump pulse (1 − *R*). By increasing *θ*_pump_, the 1 − *R* was enhanced a maximum of about 2.6 times. Therefore, all Δ*R*/*R* signals have been normalized according to the change in the 1 − *R* of the pump pulse.

**Figure 2:**
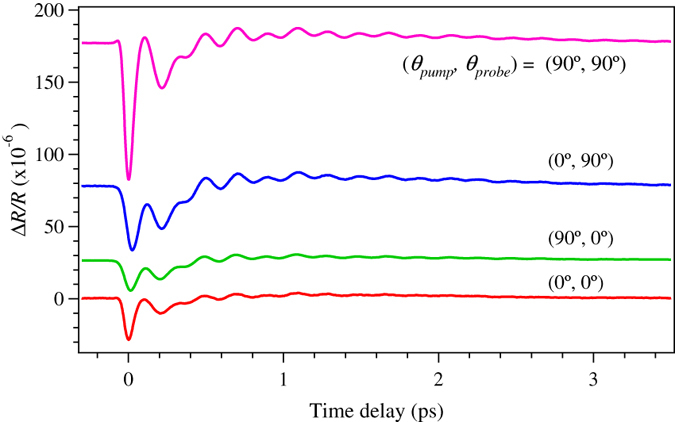
Time-domain pump-probe signals. Time-resolved Δ*R*/*R* signals for (0°, 0°), (0°, 90°), (90°, 0°) and (90°, 90°) pump-probe configurations.

In Fig. 2, Δ*R*/*R* signals obtained for the (0°, 0°), (0°, 90°), (90°, 0°) and (90°, 90°) pump-probe configurations are shown. The abrupt decrease in the signal due to the electronic response is followed by an oscillation pattern due to coherent phonons and was observed for all polarization combinations. The oscillations persist for a few picoseconds and the observed complex oscillatory patterns suggest that the signals consist of multiple coherent phonon modes. Although the effect of the polarization-dependent absorption change has been corrected for, a clear polarization dependence in both the initial electronic drop and oscillation amplitude is observed. In particular, the (90°, 90°) signal exhibits a large response compared with the other three signals. Thus, the coherent phonons as well as the electronic response of iPCM was found to be affected by the values of the *θ*_pump_ and *θ*_probe_.

**Figure 3:**
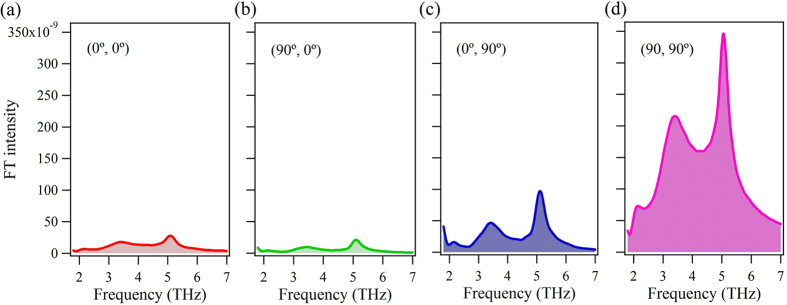
Frequency-domain pump-probe signals. FT spectra for (0°, 0°) (**a**), (90°, 0°) (**b**), (0°, 90°) (**c**) and (90°, 90°) (**d**) configurations obtained from the time-domain signals.

Figure 3 shows Fourier transformed (FT) spectra obtained from the Δ*R*/*R* signals shown in Fig. 2 after subtracting the electronic background by fitting with a combination of exponential decay functions. All spectra exhibit a relatively low intensity mode at 2.1 THz as well as two dominant modes at 3.4 and 5.1 THz. The phonon frequencies of these three modes are basically consistent with previously reported values^20^. The assignment of phonon modes in iPCM remains controversial, the 2.1 THz and 5.1 THz modes can be assigned to two different *A*_1_ modes in the Sb_2_Te_3_ sub-layers while the 3.4 THz mode may be a *E*_*g*_ mode in the Sb_2_Te_3_ sub-layers or the *A*_1_ mode in the GeTe sub-layers^20^,^21^,^22^,^23^,^24^. As seen in Fig. 3(d), the (90°, 90°) configuration leads to a significant enhancement of the FT intensity of the three modes yet the spectral shape remains basically unchanged.

**Figure 4:**
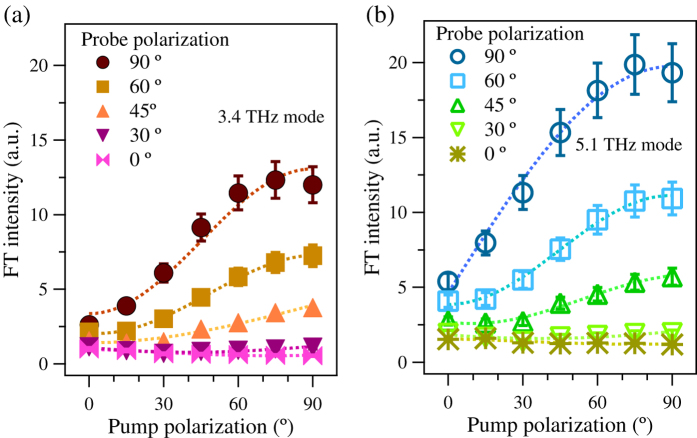
FT intensity for the various pump and probe polarization. Pump polarization dependence of FT intensity of (**a**) lower and (**b**) higher frequency modes for various probe polarization angles. The doted lines are guides to the eye.

Figure 4 shows the *θ*_pump_ dependence of the FT intensity of the 3.4 THz and 5.1 THz modes for *θ*_probe_ = 0°, 30°, 45°, 60° and 90°. Here, the FT intensity of the 3.4 THz mode obtained with (0°, 0°) polarization combination is normalized to be unity and the relative intensities are presented. Both the 3.4 and 5.1 THz modes exhibit similar trends. When *θ*_probe_ is small, the *θ*_pump_ dependence is not significant. However, with increasing *θ*_probe_, the effect of *θ*_pump_ becomes prominent. When *θ*_probe_ is large, the FT intensities significantly increase with increasing *θ*_pump_. Note that we have excluded the 2.1 THz mode from the discussion because the electronic background in the low frequency region interferes with characterization of this low frequency phonon mode. In addition to the phonon intensity variations, the spectral width of the 3.4 THz mode varies depending on the relative angle between the pump and probe pulses. In the parallel pump-probe polarization condition (0°, 0°) and (90°, 90°), the width of the mode is wide compared with that in observed in orthogonal pump-probe polarization conditions (90°, 0°) and (0°, 90°). A peculiar observation is that the pump polarization dependence does not appear when the probe polarization angle is small.

## Discussion

To clarify the origin of the (*θ*_pump_, *θ*_probe_) dependent enhancement of coherent phonon intensity, both coherent phonon generation and detection mechanisms should be considered. As mentioned above, ISRS, DECP and ultrafast screening of the SC field have been proposed as generation mechanisms for coherent phonons. In ISRS, phonons are excited by Raman scattering via real or virtual excitation of electrons. In DECP, on the other hand, abrupt modification of lattice potential due to the ultrafast generation of photo-electrons serves as the driving force. This mechanism is especially relevant for surfaces of polar semiconductors where the lattice potential can be modified by ultrafast screening of the SC field in a manner similar to DECP; this mechanism can act as a driving force for longitudinal optical phonons. In all three cases, the phonons possess coherency and become coherent phonons when the excitation pulse duration, which is directly relevant to the generation time of phonons, is sufficiently shorter than the oscillation period of the phonon modes. For the current experimental conditions in which electrons are excited across a small band gap by 1.55 eV laser irradiation, both resonant-type ISRS and DECP are applicable to the generation process of coherent phonons in iPCM. Furthermore, the ultrafast screening of the SC field has also been proposed as a generation mechanism in epitaxial Ge_2_Sb_2_Te_5_ films^24^.

If the dominant mechanism of coherent phonon generation is ISRS, the polarization dependence on Δ*R*/*R* due to phonon oscillations can be estimated based on the theory of Raman scattering. By means of Supplementary Eq. (S1), however, it was not possible to reproduce the peculiar polarization dependent behavior in which there was almost no pump polarization dependence when the *θ*_probe_ was small. If the generation mechanism of coherent phonon is instantaneous screening of the SC field, no pump polarization dependence on coherent phonon intensity is expected because only the density of photo-excited carriers is important for this mechanism^25^,^26^. Hence, ISRS and ultrafast screening of the SC field can be excluded from being the main origin of the polarization angle dependence although these mechanisms may contribute to the non-dominant and polarization-independent component of the coherent phonons. With respect to the DECP mechanism, instead, the anisotropic phonon behavior may be explained by anisotropy in the electron-phonon interaction, especially when it occurs immediately after photo-excitation. As theoretically predicted^13^, the distribution of photo-induced carriers, which is strongly governed by the direction of the applied electric field, determines the direction of the displacement of the lattice^8^,^27^,^28^. Indeed, the intensity of the Raman signal is very small in Ge-Sb-Te materials^29^ and this result implies that the Raman cross section in Ge-Sb-Te materials is not large enough for the Raman process to be the dominant generation mechanism. Alternatively, the inherently anisotropic multi-layered hexagonal iPCM structure may accentuate an anisotropic optically-induced lattice response. Since the laser pulse used in the current measurement lasts several tens of femtoseconds (30 fs at full width at half maximum), it affects not only the ground state but also the photo-excited state. Therefore, in addition to the anisotropic electron-phonon interaction, the existence of the pump-pulse field may also contribute to directional polarizability change. The details of this possible mechanism will be unveiled by further studies.

**Figure 5:**
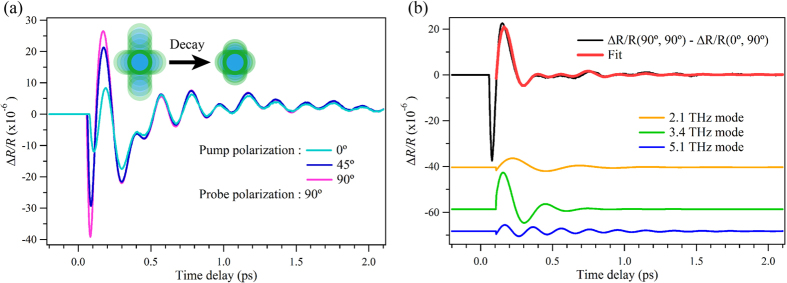
Comparison of early time stage of coherent phonon oscillations obtained with different pump angles and summary of anisotropic phonon decay. (**a**) Δ*R*/*R* signals for (0°, 90°), (45°, 90°) and (90°, 90°) obtained by subtracting the carrier responses. The inset shows a schematic illustration of the decay of the anisotropic phonon oscillations. (**b**) The difference between the pink and light blue curves of (**a**). The red curve shows a fit obtained using eq. (1). The orange, green and blue curves are the decomposed curves of the fit of the 2.1 THz, 3.4 THz and 5.1 THz modes, respectively.

To better understand the details of the anisotropy, we calculated the oscillatory components of the Δ*R*/*R* signals for (0°, 90°), (45°, 90°) and (90°, 90°) data by subtracting the non-oscillatory electronic response using double exponential function fitting as shown in Fig. 5(a). It was found that the pump polarization angle dependence emerges predominantly in the first two cycles of the oscillation pattern and the profiles of later oscillation are nearly identical. This tendency also cannot be explained by the theory of Raman scattering. Since the *θ*_probe_ was fixed at 90°, these signals reflect the change in the phonon-induced polarizability along the particular direction which couples with 90°-polarized light. When the pump pulse is polarized at 90°, the induced electronic distribution (namely the driving force of coherent phonons) preferentially acts along a specific direction and causes anisotropic phonon oscillations that interact with the 90°-polarized probe pulse. By using a 90°-pump pulse and a 90°-probe pulse, both the anisotropic and isotropic component of coherent phonon oscillations can be detected. In contrast, the coherent phonons induced by 0°-pump pulse possess different anisotropic oscillation components and isotropic components are dominantly detected by a 90°-probe pulse. Hence, the difference between the (90°, 90°) and (0°, 90°) signals should correspond to the anisotropic component of the coherent phonons which decays faster than the isotropic component due to the symmetry of crystal as shown in the inset of Fig. 5(a).

To obtain insight into this directional phonon decay, the anisotropic component of the coherent phonons was extracted by calculating the difference between 90°-pump and 0°-pump signals as shown in Fig. 5(b). The trace was well fitted with a combination of three damped harmonic oscillations,


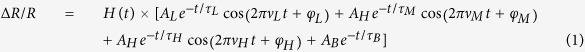


where *A*, *ω*, *τ* and *φ* are the amplitude, the frequency, the dephasing time and the initial phase of the dominant two coherent phonon modes and the subscripts *L*, *M*, *H* and *B* represent the lower, the middle and the higher frequency modes and background component. *H*(*t*) is the Heaviside step function convoluted with a Gaussian to account for the finite time resolution. The obtained values of the directional phonon decay times for the lower, the middle and the higher modes were 0.28, 0.15 and 0.47 ps, respectively. The relatively short anisotropic decay time observed for the 3.4 THz mode is likely to be related to the symmetry of phonon mode. Within the current study, both the 3.4 and 5.1 THz modes exhibited a similar pump and probe polarization dependence although these are different modes. This result can be attributed to the disturbance of the crystal. Even for an epitaxial Ge_2_Sb_2_Te_5_ fabricated on a GaSb(001) substrate, the symmetry of crystal is distorted to some extent due to the presence of intrinsic vacancies and Shalini *et al.* found by extensive investigation of the coherent phonons that the phonon modes were significantly modified and became unspecific^24^. Polycrystalline samples are expected to show more ambiguous phonon properties than epitaxial samples and thereby the two different modes likely show a similar trend.

In conclusion, we have investigated optically induced coherent phonons in the (GeTe)_2_/(Sb_2_Te_3_)_4_ interfacial phase change memory material by employing optical pump-probe time-domain spectroscopy with various polarization angles of linearly-polarized pump and probe pulses. Almost no pump polarization dependence was observed when the polarization angle of the probe pulse was perpendicular to the *c*–axis, while the dependence became significant with increasing in the field component parallel to the *c*–axis. By comparing the time-domain signals obtained with different pump polarizations and the same probe polarization, we found the difference only occurred in the early time stage of the phonon oscillations. Since these results can not be explained by the theory of Raman scattering, we attribute the origin of the polarization dependence to anisotropically-induced transient lattice oscillations which can be excited and detected by optical pulses with a specific polarization angle. The excitation of anisotropic lattice response can be a powerful means for further development of ultrafast control of matter. Our technique will be combined with other ultrafast phonon-induced lattice control methods^5^,^6^,^7^ to improve their efficiency.

## Methods

### iPCM sample

The [(GeTe)_2_/(Sb_2_Te_3_)_4_]_8_ sample, which consisted of alternately grown 1.0 nm GeTe and 4.0 nm Sb_2_Te_3_ layers, was grown on the Si (100) substrate covered with 5 nm-thick Sb_2_Te_3_ initial layer that ensured fabrication of a highly ordered superlattice^30^,^31^. The substrate temperature was set to 230 °C during the growth. A 20-nm thick ZnS-SiO_2_ protection layer was deposited on top of the sample to prevent oxidation. Every layer was deposited by RF magnetron sputtering using alloy targets.

### Coherent phonon spectroscopy

Optical pump-probe measurements were carried by employing a femtosecond Ti:Sapphire laser oscillator. Time-resolved reflectivity change of the probe pulses (Δ*R*/*R*) was recorded as a function of pump-probe time delay at room temperature utilizing a near-infrared optical pulse of 30 fs duration and a center wavelength of 830 nm at a repetition rate of 80 MHz. The pulse duration at the sample was optimized by a pair of dispersion-compensating prisms to preserve the time resolution of the system 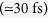. The linearly polarized pump and probe pulses were focused with a 33.85 mm focal length 60°-off-axis parabolic mirror at 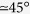 and 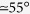 with respect to the sample normal, respectively. These incident angles were chosen to enable to apply electric field along both in-plane and out-of-plane directions yet not to worsen the signal-to-noise ratio due to the enlargement of size of focused spots. The fluence of the pump pulse was kept at 756 *μ*J cm^−2^, from which we estimated a temperature rise of only 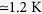 by means of the two-temperature model^32^,^33^, so that the increase in the sample temperature due to laser irradiation could be neglected. Moreover, we have confirmed that repetitive laser irradiation does not cause damage and phase change on the sample surface. The polarization angles of the pump and probe pulses were rotated using 1.3 mm-thick zero-order quartz half-wave plates. The polarization dependence of the optical reflectivity (*R*) of the pump pulse was obtained by a static reflectivity measurement and the obtained 1 − *R* data is shown in Fig. 1(b). On the other hand, the *θ*_probe_ dependence of optical absorption for probe pulse can be neglected because Δ*R*/*R* does not depend on the probe intensity and nonlinear effect are not expected for the current probe power.

## Additional Information

**How to cite this article**: Makino, K. *et al.* Anisotropic lattice response induced by a linearly-polarized femtosecond optical pulse excitation in interfacial phase change memory material. *Sci. Rep.* **6**, 19758; doi: 10.1038/srep19758 (2016).

## Supplementary Material

Supplementary Information

## References

[b1] Först M. *et al.* Nonlinear phononics as an ultrafast route to lattice control. Nature Phys. 7, 854–856 (2011).

[b2] Rini M. *et al.* Control of the electronic phase of a manganite by mode-selective vibrational excitation. Nature 449, 72–74 (2007). 17805291 10.1038/nature06119

[b3] Lee J. D. & Hase M. Coherent optical control of the ultrafast dephasing of phonon-plasmon coupling in a polar semiconductor using a pulse train of below-band-gap excitation. Phys. Rev. Lett. 101, 235501 (2008). 19113565 10.1103/PhysRevLett.101.235501

[b4] Kim K. W. *et al.* Ultrafast transient generation of spin-density-wave order in the normal state of BaFe_2_As_2_ driven by coherent lattice vibrations. Nature Mater. 11, 497–501 (2012). 22484832 10.1038/nmat3294

[b5] Dumitrică T., Garcia M. E., Jeschke H. O. & Yakobson B. I. Selective cap opening in carbon nanotubes driven by laser-induced coherent phonons. Phys. Rev. Lett. 92, 117401 (2004). 15089164 10.1103/PhysRevLett.92.117401

[b6] Liebig C. M., Wang Y. & Xu X. Controlling phase change through ultrafast excitation of coherent phonons. Optics Exp. 18, 20498–20504 (2010). 10.1364/OE.18.020498 20940942

[b7] Makino K., Tominaga J. & Hase M. Ultrafast optical manipulation of atomic arrangements in chalcogenide alloy memory materials. Optics Exp. 19, 1260–1270 (2011). 10.1364/OE.19.001260 21263667

[b8] Makino K., Tominaga J., Kolobov A. V., Fons P. & Hase M. Polarization dependent optical control of atomic arrangement in multilayer Ge-Sb-Te phase change materials. Appl. Phys. Lett. 101, 232101 (2012).

[b9] Yan Y. X., Gamble E. B. & Nelson K. A. Impulsive stimulated scattering: General importance in femtosecond laser pulse interactions with matter, and spectroscopic applications. J. Chem. Phys. 83, 5391–5399 (1985).

[b10] Merlin R. Generating coherent THz phonons with light pulses. Solid State Commun. 102, 207–220 (1997).

[b11] Zeiger H. J. *et al.* Theory for displacive excitation of coherent phonons. Phys. Rev. B 45, 768–778 (1992). 10.1103/physrevb.45.768 10001117

[b12] Cho G. C., Kütt W. & Kurz H. Subpicosecond time-resolved coherent-phonon oscillations in gaas. Phys. Rev. Lett. 65, 764–766 (1990). 10043013 10.1103/PhysRevLett.65.764

[b13] Murray E. D. & Fahy S. First-principles calculation of femtosecond symmetry-breaking atomic forces in photoexcited bismuth. Phys. Rev. Lett. 114, 055502 (2015). 25699453 10.1103/PhysRevLett.114.055502

[b14] Simpson R. E. *et al.* Interfacial phase-change memory. Nature Nanotech. 6, 501–505 (2011). 10.1038/nnano.2011.96 21725305

[b15] Wutting M. & Yamada N. Phase-change materials for rewriteable data storage. Nature Mater. 6, 824–832 (2007). 17972937 10.1038/nmat2009

[b16] Tominaga J., Simpson R. E., Fons P. & Kolobov A. V. Electrical-field induced giant magnetoresistivity in (non-magnetic) phase change films. Appl. Phys. Lett. 99, 152105 (2011).

[b17] Bang D. *et al.* Mirror-symmetric magneto-optical kerr rotation using visible light in [(GeTe)_2_(Sb_2_Te_3_)_1_]_*n*_ topological superlattices. Sci. Rep. 4, 5727 (2014). 25030304 10.1038/srep05727 PMC4101470

[b18] Sa B., Zhou J., Sun Z., Tominaga J. & Ahuja R. Topological insulating in GeTe/Sb_2_Te_3_ phase-change superlattice. Phys. Rev. Lett. 109, 096802 (2012). 23002870 10.1103/PhysRevLett.109.096802

[b19] Tominaga J., Kolobov A. V., Fons P., Nakano T. & Murakami S. Ferroelectric order control of the dirac-semimetal phase in GeTe-Sb_2_Te_3_ superlattices. Adv. Mater. Interfaces. 1, 1300027 (2014).

[b20] Makino K. *et al.* Coherent phonon study of (GeTe)_2_(Sb_2_Te_3_)_*m*_ interfacial phase change memory materials. Appl. Phys. Lett. 105, 151902 (2014).

[b21] Först M. *et al.* Phase change in Ge_2_Sb_2_Te_5_ films investigated by coherent phonon spectroscopy. Apll. Phys. Lett. 77, 1964–1966 (2000).

[b22] Hase M., Miyamoto Y. & Tominaga J. Ultrafast dephasing of coherent optical phonons in atomically controlled GeTe/Sb_2_Te_3_ superlattices. Phys. Rev. B 79, 174112 (2009).

[b23] Hernandez-Rueda J. *et al.* Coherent optical phonons in different phases of Ge_2_Sb_2_Te_5_ upon strong laser excitation. Appl. Phys. Lett. 98, 251906 (2011).

[b24] Shalini A. *et al.* Observation of *T*_2_-like coherent optical phonons in epitaxial Ge_2_Sb_2_Te_5_/GaSb(001) films. Sci. Rep. 3, 2965 (2013). 24129388 10.1038/srep02965 PMC3797426

[b25] Yee K. J., Lim Y. S., Dekorsy T. & Kim D. S. Mechanisms for the generation of coherent longitudinal-optical phonons in gaas/AlGaAs multiple quantum wells. Phys. Rev. Lett. 86, 1630–1633 (2001). 11290210 10.1103/PhysRevLett.86.1630

[b26] Mizoguchi K. *et al.* Characterization of terahertz electromagnetic waves from coherent longitudinal optical phonons in GaAs/AlAs multiple quantum wells. J. Appl. Phys. 100, 103527 (2006).

[b27] Pfeifer T., Kütt W., Kurz H. & Scholz R. Generation and detection of coherent optical phonons in germanium. Phys. Rev. Lett. 69, 3248–3251 (1992). 10046768 10.1103/PhysRevLett.69.3248

[b28] Scholz R., Pfeifer T. & Kurz H. Density-matrix theory of coherent phonon oscillations in germanium. Phys. Rev. B 47, 16229–16236 (1993). 10.1103/physrevb.47.16229 10006045

[b29] Andrikopoulos K. S., Yannopoulos S. N., Kolobov A. V., Fons P. & Tominaga J. Raman scattering study of gete and Ge_2_Sb_2_Te_5_ phase-change materials. J. Phys. Chem. Solid 68, 1074–1078 (2007).

[b30] Simpson R. E., Fons P., Kolobov A. V., Krbal M. & Tominaga J. Enhanced crystallization of GeTe from an Sb_2_Te_3_ template Appl. Phys. Lett. 100, 021911 (2012).

[b31] Saito Y., Fons P., Kolobov A. V. & Tominaga J. Self-organized van der waals epitaxy of layered chalcogenide structures. Phys. Stat. Sol. (b), 252, 2151–2158 (2015).

[b32] Kaganov M. I., Lifshitz I. M. & Tanatarov L. V. J. Relaxation between electrons and crystalline lattice. Sov. Phys. JETP 4, 173–178 (1957).

[b33] Allen P. B. Theory of Thermal Relaxation of Electrons in Metals. Phys. Rev. Lett. 59, 1460–1463 (1987). 10035240 10.1103/PhysRevLett.59.1460

