# Supplementary material for: Anisotropic lattice response induced by a linearly-polarized femtosecond optical pulse excitation in interfacial phase change memory material
Source: Sci Rep. 2016 Jan 25;6:19758. doi: 10.1038/srep19758 (PMC4726132; doi:10.1038/srep19758)
Supplement: Supplementary Information [file srep19758-s1.pdf]

Supplementary Material for "Anisotropic lattice response induced by linearly-polarized femtosecond optical pulse excitation in interfacial phase change memory material"

Kotaro Makino[1,2,*], Yuta Saito[1,2], Paul Fons[1,2], Alexander V. Kolobov[1,2], Takashi Nakano[1,2], Junji Tominaga[1,2], and Muneaki Hase[2,3]

[1]Nanoelectronics Research Institute, National Institute of Advanced Industrial Science and Technology (AIST), Tsukuba Central 5, 1-1-1 Higashi, Tsukuba 305-8562, Japan

[2]CREST, JST, 4-1-8 Honcho, Kawaguchi, Saitama, 332-0012, Japan

[3]Division of Applied Physics, Faculty of Pure and Applied Sciences, University of Tsukuba, 1-1-1 Tennodai, Tsukuba 305-8573, Japan

*k-makino@aist.go.jp

Raman scattering in our iPCM sample

In the framework of Impulsive Stimulated Raman Scattering (ISRS), the $\Delta R/R$ due to a phonon oscillation can be expressed as,

$$\Delta R/R \propto \left|\left((E_{\mathrm{pump}}^{t} M E_{\mathrm{probe}}\right)\right|^2$$

Here, $\boldsymbol{M}$ represents the Raman tensor, while E_{pump} and, E_{probe} are the polarization vectors of the pump and probe pulses, respectively. In our structural model of the iPCM sample based on *ab-initio* calculations and cross-sectional high-resolution transmission electron microscope observations, $\mathrm{P}\bar{3}\mathrm{m1}$ symmetry can be imposed and the point group is D_{3d} in which the A_{1g} and E_g optical phonon modes are Raman active. Under D_{3d} symmetry, the Raman tensors for A_1, E_x and E_y modes are

$$\begin{pmatrix} a & \cdot & \cdot \\ \cdot & a & \cdot \\ \cdot & \cdot & b \end{pmatrix}, \begin{pmatrix} c & \cdot & \cdot \\ \cdot & -c & d \\ \cdot & d & \cdot \end{pmatrix}, \begin{pmatrix} \cdot & -c & -d \\ -c & \cdot & \cdot \\ -c & \cdot & \cdot \end{pmatrix}$$

E_{pump} and E_{probe} can be written as,

$$E_{pump} \propto \begin{pmatrix} \cos(55°)\cdot\sin(\theta_{pump}) \\ \cos(55°)\cdot\cos(\theta_{pump}) \\ \sin(55°)\cdot\sin(\theta_{pump}) \end{pmatrix},$$

$$E_{probe} \propto \begin{pmatrix} \cos(45°) \cdot \sin(\theta_{probe}) \\ \cos(45°) \cdot \cos(\theta_{probe}) \\ \sin(55°) \cdot \sin(\theta_{probe}) \end{pmatrix},$$

Note that we have confirmed the polycrystalline fiber structure of the sample where the *a*-*b* planes are randomly oriented within the laser spots. In fact, we confirmed the absence of in-plane sample rotation dependence on coherent phonon intensity at various position of the sample under normal incident conditions (not shown). On the other hand, the *c*-axis of the iPCM was confirmed to be oriented along the sample normal direction by the x-ray diffraction measurements. Therefore, no difference between *x*- and *y*-directional signals is expected in our result and anisotropic response could be observed between *c*-axis and *a*-*b* plane.
